# A Compact Model of Ovonic Threshold Switch Combining Thermal Dissipation Effect

**DOI:** 10.3389/fnins.2021.635264

**Published:** 2021-02-09

**Authors:** Shiqing Zhang, Hui Xu, Zhiwei Li, Sen Liu, Bing Song, Qingjiang Li

**Affiliations:** College of Electronic Science and Technology, National University of Defense Technology, Changsha, China

**Keywords:** ovonic threshold switch, physical model, chalcogenides, thermal dissipation effect, thermal conductivity

## Abstract

Ovonic threshold switch (OTS) has received great attention in neuromorphic computing due to its support for high-density synapse array as a selector and leaky-integration-firing functions Hodgkin-Huxley neurons. However, there is no simple and complete model for device simulation and integrated circuit design, which hindered application until now. In this work, we developed a compact physical model of OTS based on the Poole-Frenkel effect accompanied by the thermal dissipation effect for the first time. The thermal dissipation effect describes the energy flow between the device and the environment so that the model is more practical. Compared with previous experiments, the numerical results fairly fitted the electrical characteristics, demonstrating the model validity. In addition, the relation of the device performance with material and structure was deduced, which can facilitate optimizing the OTS device. The model will be useful for device design and implemented with high speed for simplicity.

## Introduction

As a promising nano device, ovonic threshold switch (OTS) based on chalcogenide plays an important role in neuromorphic computing (Tuma et al., 2016). OTS possesses small features, simple structure, high switching speed and low energy consumption, which can be fit for novel applications (Song et al., 2019; Laudato et al., 2020; Raty and Noé, 2020). For example, it can help to relieve the sneak current that flows through the unselected cell in the memristor synapse array when integrated with the memristor (Laudato et al., 2020; Molas et al., 2020). Besides, signal transmission in neurons is mediated by the dynamic permission or prevention of sodium and potassium ion channels (Pickett et al., 2013; Yi et al., 2018). OTS, as two-terminal device, has been known to exhibit current-controlled negative differential resistance for a long period of time, referred to as threshold switching. This phenomenon can simulate the neural signal transmission, so it is progressively becoming one of the prime candidates to manufacture a Hodgkin-Huxley neuron (Li et al., 2014; Tuma et al., 2016).

In the meantime, a compact accurate model of OTS is lacking application. From the 1970s, different models and mechanisms have been proposed to account for the threshold switching characteristic (Nardone et al., 2012). Ielmini and Zhang (2007) demonstrated an analytical model for phase change memory which utilizes chalcogenide like OTS. It explained the threshold switching based on trap-limited conduction, non-equilibrium carrier distribution and non-uniformity of electric field mechanism. Moreover, a trap-to-band hopping transport process (Wang et al., 2010; Shen et al., 2014) and thermally assisted hopping effect (Piccinini et al., 2012; Jacoboni et al., 2013), etc. are proposed to refine the model in detail. Nevertheless, there are two facts needed to be further studied. First, most of the previous models only considered increasing voltage sweeping and the process of back sweeping remains poorly understood. Besides, the heat flow between the device and the external environment has been neglected, and the thermal effect is important for chalcogenide (Huang and Hsieh, 2015).

Based on the above research, the impact of thermal effect on OTS conduction mechanism is further studied. “Physical Switching Model” section presents a compact and complete bidirectional threshold switching selector physical synthesis model based on Poole-Frenkel combining thermal dissipation effect. Subsequently, some important structural and material parameters is extracted from the modeling results. The simulation results of different devices and the effects of these parameters on threshold voltage, selectivity, off-state current, etc., were discussed in “Model Validation” section. At last, according to the analysis above, a high threshold voltage and high selectivity sample is manufactured to verify the validity of the model in “Application of Model” section.

## Physical Switching Model

### Analysis of Physical Phenomenon

Chalcogenide glass, as a kind of amorphous semiconductor material, has many defects in the band gap, implying the existence of a number of trap states (Ovshinsky, 1968). Much research has indicated that the threshold switching is a pure electron process (Jacoboni et al., 2013; Chen et al., 2016; Noe et al., 2020). If the conduction was cause by a pure electron process, carriers would tunnel between traps. In fact, however, chalcogenide glass is a temperature sensitive material and the pure hopping is mainly effective in the low-temperature range. As for thermally effected tunneling, its dependence was shown on temperature and field (Karpov and Niraula, 2019). Therefore, with the rise of temperature, we believe that the energy produced by heat will have a positive influence on the mobile carrier concentration. The rapid accumulation of the mobile carriers contributes to the current mutation. The phenomenon is supposed to cause the temperature and field changes during the pure electron process. Simultaneously, the position of the threshold switching may drift. For these reasons, it is reasonable to take the effect of heat energy into account. Nevertheless, in the previous models, the final temperature of the device can even reach tens of thousands of degrees centigrade, which is obviously unrealistic. The flow of energy occurs not only inside the device, but also in the external environment. The thermal dissipation effect is able to describe this phenomenon accurately. Therefore, the thermal dissipation effect is taken into the physical model of OTS for the first time in our work.

### Mathematical Description of Model

First of all, in order to simplify the whole physical process, the physical quantities of interest are considered merely in equilibrium condition which denotes the final state of every stimuli, compared to previous reports (Jacoboni et al., 2017). The time impact will be ignored since the switching time of OTS device is short enough (ns/ps order of magnitude). Therefore, the device dynamics is assumed to be one-dimensional: the characteristics of the device are functions with respect to the longitudinal coordinate *x*.

Furthermore, a single energy level assumption is adopted here for the mobile states. The carriers are divided into two parts that can occupy two levels with energy values **E_T_** and **E_B_**. Here, **E_T_** means the energy of trapped carriers, while **E_B_** means the energy of mobile carriers. The difference between *E_B_* and **E_B_** determines the energy required for the hopping of carriers. For simplification, **E_T_** = **0**, **E_B_** = △. Besides, **g_T_** corresponds to the density of states in level ***E*_*T*_** and **g_B_** corresponds to the density of states in level **E_*B*_**.

Moreover, the electric field is supposed to be uniform when voltage is applied to both ends of the device. According to the Poole-Frenkel theory (Ielmini and Zhang, 2007), the electric field induces the band bending and the energy required for the hopping of carriers changing.

(1) $$\Delta \left(x\right)={\text{E}}_{B}-{\text{E}}_{T}-\sqrt{\frac{\text{q}F\left(x\right)}{\pi \varepsilon \varepsilon_{0}}}$$

The number of carriers in the device consist of the mobile states **n_B_** and trapped states **n_T_**:

(2) $$\text{n}\left(x\right)={\text{n}}_{T}\left(x\right)+{\text{n}}_{B}\left(x\right)$$

Thereinto, only carriers in the mobile states can move so that the density of carriers in the mobile states is given by Eq. (3) based on Maxwellian distributions:

(3) $${\text{n}}_{B}\left(x\right)=\frac{\text{n}\left(x\right)}{1+\Gamma \text{e}xp\left(\frac{\Delta \left(x\right)}{k_{B}T\left(x\right)}\right)}$$

Here, **k_B_** is Boltzmann constant, **Γ** is the ratio of density of states **g_T_** to **g_B_**. The mobile carrier concentration has exponential relation with the width of band gap and temperature, contributing to the sudden change of current.

The Eq. (4) shows the calculation equation of current density and it comprises two parts, namely drift current, and diffusion current.

(4) $$\text{j}\left(x\right)=q\left[{\text{n}}_{B}\left(x\right)\mu \text{F}\left(x\right)-{\text{D}}_{B}\frac{{\text{n}}_{B}\left(x\right)}{{\text{L}}_{n}}\right]$$

The former is the drift current and the latter is the diffusion current. Here, **D_B_** is the diffusion coefficient of the mobile electrons. It assumes here to be given by the equilibrium Einstein relation: **D_B_** = μ**k_B_**/**q**, with **L_n_** as the diffusion thickness of the mobile electrons, assumed here to be given by ${\text{L}}_{n}=\sqrt{{\text{D}}_{B}\tau },$ with τ the carrier’s life.

The temperature and the thermal energy are of vital importance in our model. For the description of the thermal effect, we assume all the current flowing through the device leads to a temperature increased by Joule heating (Jeon et al., 2016; Al-Mamun and Orlowski, 2020). Furthermore, due to the time impact having been ignored, we assume that when applied voltage changes every time, the device has enough time to reach equilibrium. Therefore, the temperature distribution is uniform at every applied voltage.

Based on energy balance equation, the energy pumped by the field transforms to the space variation of the current and the processing of electron temperature relaxing which, in this case, is described by a temperature relaxation time **τ_T_** (Jacoboni et al., 2017):

(5) $$\text{q}jF-\Delta \left(x\right)\frac{\text{j}\left(x\right)}{{\text{L}}_{n}}-\text{n}\frac{{\text{k}}_{B}\text{T}-{\text{k}}_{B}{\text{T}}_{0}}{\tau_{T}}=0$$

The Eq. (5) can be deformed to:

(6) $${\text{T}}_{generation}\left(x\right)={\text{T}}_{0}+\frac{\tau_{T}}{\text{n}\left(x\right)}\left[\text{j}\left(x\right)\text{F}\left(x\right)-\Delta \left(x\right)\frac{\text{j}\left(x\right)}{{\text{L}}_{n}}\right]$$

In fact, only the effect of internal temperature is considered in previous models, resulting in inaccuracy. In actual operation, the heat generated will eventually release to the environment, leading to the following with the temperature difference denoting the state variable. The thermal dissipation effect describes the dynamics of the temperature state variable **T** based on Newton’s law of cooling, is introduced into the model (Pickett and Williams, 2012; Slesazeck et al., 2015):

(7) $${\text{T}}_{dissipation}\left(x\right)=\frac{{\text{R}}_{m}{\text{i}}_{m}^{2}-\Gamma_{th}\Delta \text{T}}{{\text{C}}_{th}}$$

Here, **C**_**t***h*_ is the thermal capacitance and **Γ_t*h*_** is the thermal conductance. The first term in Eq. (7) depicts the temperature increase due to Joule heating, where **R_m_** is the device resistance depending on the current and voltage of the previous state. Hence, Eq. (6) is developed into:

(8) $$\text{T}\left(x\right)={\text{T}}_{generation}\left(x\right)-{\text{T}}_{dissipation}\left(x\right)$$

At the initial time, all quantities are known and take their equilibrium values: (a) △(**1**) = **E_B_**; (b) **F**(**1**) = **0**; (c) **T**(**0**) = **T_0_**. **T_0_** indicates the room temperature. Each variable above receives the result in terms of the other unknowns so that the equations must be solved numerically. The iterative algorithm is employed with the following procedure:

(i) △(**x**) can be gained from Eq. (1);
(ii) from Eq. (7) one gets the updated value for **T**_**d***issipation*_(**x**);
(iii) Eqs. (6) and (8) provide the updated value for **T**(**x**);
(iv) the knowledge of **T**(**x**) allows for the updated evaluation of **n_B_**(**x**) from Eq. (3);
(v) Once △(**x**), **T**(**x**) and **n_B_**(**x**) are known, the updated value for **j**(**x**) is obtained by solving Eq. (5).

## Model Validation

### Simulation Verification

In this part, numerical results are presented and discussed based on the above theory first.

**FIGURE 1:**
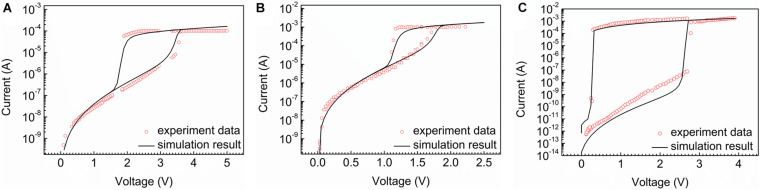
The simulation result (black line) and the experiment data (red dots). **(A)** High threshold voltage and low selectivity sample. **(B)** Low threshold voltage and low selectivity sample. **(C)** High selectivity high threshold voltage sample.

According to the reference parameters, the validity of the whole model is verified by matching the characteristics of the experiment devices. Three types of typical devices are selected for simulation verification, namely, high voltage and low selectivity devices, low voltage and low selectivity devices, and high voltage and high selectivity devices. Figure 1A shows the outcome of the simulation result of AsTeGeSiN-based 40 nm OTS selector device (Lee et al., 2012; Kim S. et al., 2013). The device exhibits the performance of the high threshold voltage and low selectivity. The device parameters will be tested and described by the physical and geometrical parameters list in Table 1.

**TABLE 1:** Physical parameters used in the simulations, unless differently specified in the text (Kim S.D. et al., 2013).

Symbol	Definition	Value	Units
E_*T*_	Energy level of traps	0	eV
E_*B*_	Energy level of mobile states	0.42	eV
Δ	E_*B*_ - E_*T*_	0.42	eV
g_*T*_	DoS of trap states		eV^–1^
g_*B*_	DoS of mobile states		eV^–1^
Γ	g_*T*_/g_*B*_	2.25 × 10^–3^	
ε	Relative permittivity of the material	15	
μ	Mobility of mobile electrons	1 × 10^–3^	m^2^ (Vs)^–1^
n_0_	Equilibrium electron concentration	2 × 10^22^	m^–3^
τ_*T*_	Temperature relaxation time	1 × 10^–14^	s
τ	Carrier life	10 × 10^–6^	s
L	Device thickness	40 × 10^–9^	m
T_0_	Room temperature	300	K
C_*th*_	Thermal capacitance	12 × 10^–6^	J K^–1^
Γ_*th*_	Thermal conductance	6 × 10^–8^	W K^–1^

The actual current–voltage (I–V) property of the device, signed as a red dot, shows that the current occurs a sudden rise when the applied voltage reaches V_*th*_ (3.3 V). Then, when the voltage decreases back to the holding voltage V_*h*_ (1.8 V), the sudden drop of current occurs, accounting for the cell back to the initial state.

In addition, devices based on different material systems are employed to verify the correctness of our model, shown in Figures 1B,C. Figure 1B shows a low threshold voltage and low selectivity sample based on GeTe_6_ with 20 nm thickness (Velea et al., 2017). It performs a low threshold voltage characteristic at around 1.7 V and holds voltage at 1.2 V and low selectivity (∼10^2^). Figure 1C presents a multilayer OTS device based on SeSb/SeSbGeN and it demonstrates high selectivity characteristics (∼10^6^) with high threshold voltage at 2.7 V (Laguna et al., 2020). The simulation results match well with the experimental data and present two processes of opening and closing at the same time, which indicates that our model is able to accord with both low voltage and high selective conditions perfectly, showing the validity of the model.

### Results and Discussion

In this section, we present and discuss results based on the above model. First, five key factors are employed as the model simulation input parameters to influence the device characteristics. They are divided into two parts, namely material parameters, including width of band gap, relative permittivity of the material, temperature relaxation time and mobility of mobile electrons, and structure parameter, namely thickness of device. Then the current characteristic, temperature characteristic and mobile carriers are developed as the model simulation output. At last, we focus on threshold voltage (V_*th*_), hold voltage (V_*h*_), selectivity, off-state current as the final results to depict the performance of the device.

#### Current Characteristic

**FIGURE 2:**
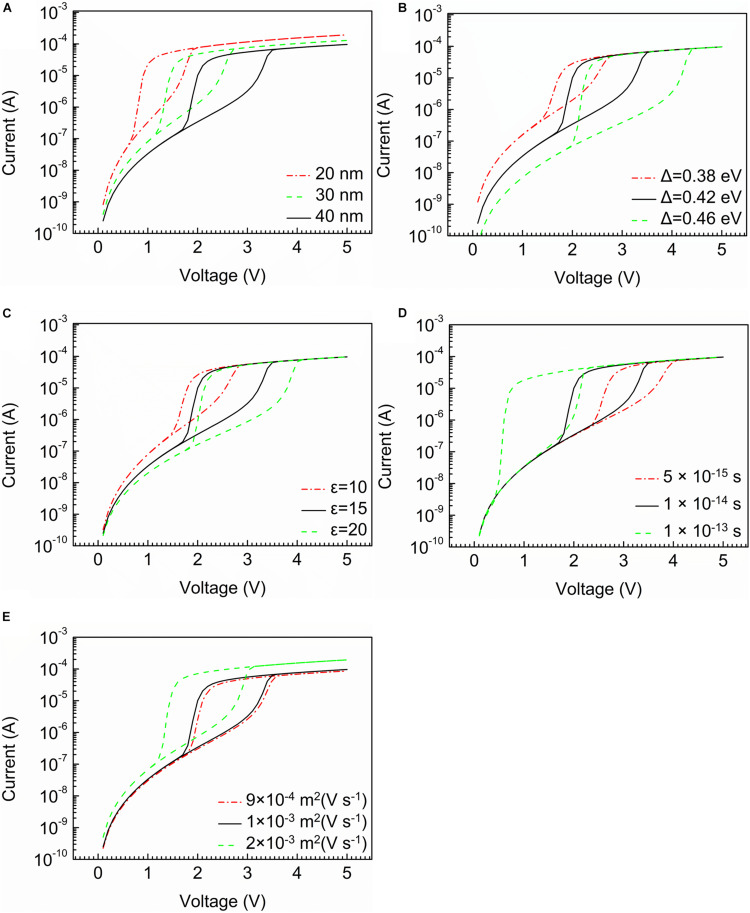
The current characteristic curves with different parameters. **(A)** Thickness of device. **(B)** Width of band gap. **(C)** Relative permittivity of the material. **(D)** Temperature relaxation time. **(E)** Mobility of mobile electrons.

The current characteristic curves with different parameters are shown in Figures 2A–F.

The structure parameter plays vital roles in the electrical characteristics of the cell, as shown in Figure 2A. When thickness of device increases from 20 to 40 nm, V_*th*_ has an obvious rise from 1.5 to 3.3 V. The trend of the holding voltage is consistent with the threshold voltage. However, the structure parameter is not able to change selectivity. Meanwhile, the off-state current decreases accompanied by the growth of the thickness of the device.

Figures 2B–E indicate the effect of the material parameters. Figure 2B shows that the width of the band gap has an influence on the current characteristic. With the width of the band gap rising from 0.35 to 0.46 eV, there is a significant rise in selectivity from 10^2^ to 10^3^. Threshold voltage increase from 2.1 to 4 V in the meantime, indicating a higher power consumption. And the off-state current also rises obviously. Figure 2C presents the relationship between relative permittivity of the material and current characteristic. It can be found that the selectivity increases from 10^2^ to 10^4^, when the relative permittivity rises from 10 to 20, and threshold voltage decreases with the decline of relative permittivity. The off-state current increases slightly with the increase of relative permittivity. Figure 2D illustrates the current characteristics influenced by temperature relaxation time. The outcomes show that the increase of temperature relaxation time results in a sharp decline in threshold voltage. The longer the temperature relaxation time is, the larger the selectivity is. However, it has no effect on off-state current. In Figure 2E, on-state current rises with a higher mobility of mobile electrons. In addition, the growth of the mobility has a positive influence on current increase, nonetheless, the influence of this factor on the selective is not obvious.

#### Mobile Carrier Concentration

**FIGURE 3:**
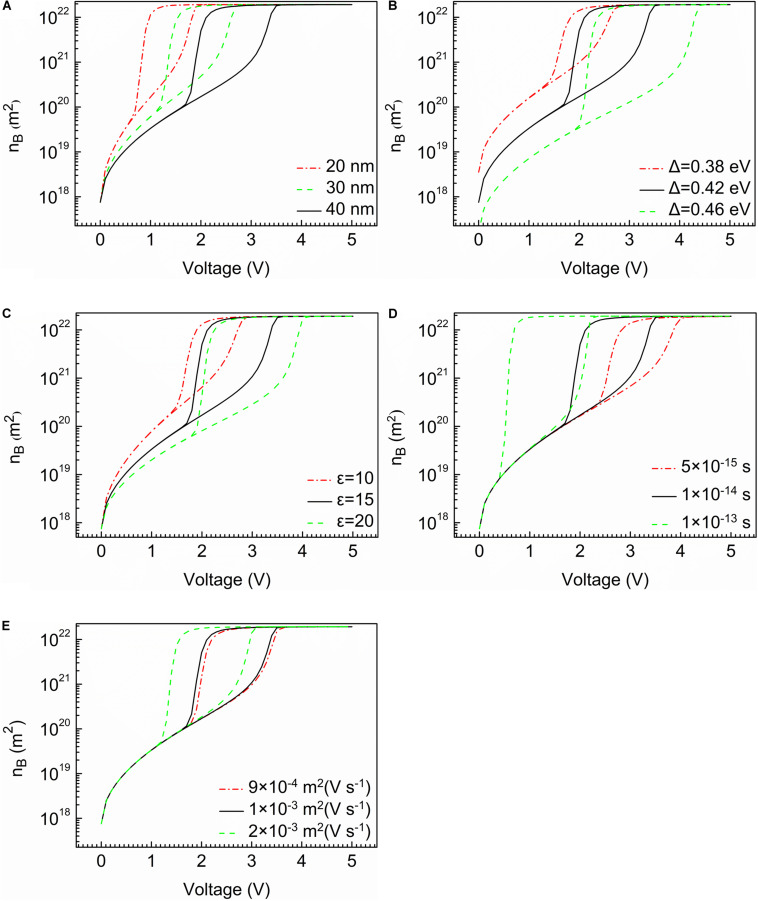
The characteristic of mobile carrier concentration curves. **(A)** Thickness of device. **(B)** Width of band gap. **(C)** Relative permittivity of the material. **(D)** Temperature relaxation time. **(E)** Mobility of mobile electrons.

The continuous lines of Figures 3A–E show the relationship between the mobile carrier concentration and voltage.

It is noteworthy that mobile carrier concentration causes sudden changes to occur at V_*th*_ and V_*h*_, respectively, similar to the current characteristic. As shown in Figure 3B, with the increase of width of band gap, mobile carrier concentration has a remarkable reduction before switching. In Figures 3D,E, the temperature relaxation time and the mobility of mobile electrons have no effect on the mobile carrier concentration before and after switching.

#### Temperature Characteristic

**FIGURE 4:**
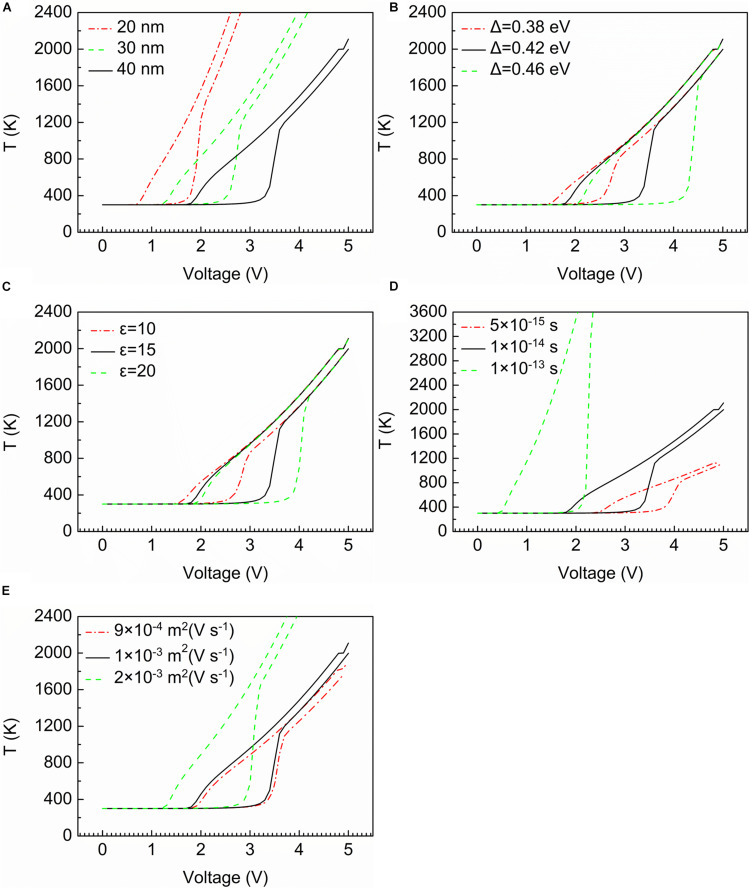
The temperature characteristic curves. **(A)** Thickness of device. **(B)** Width of band gap. **(C)** Relative permittivity of the material. **(D)** Temperature relaxation time. **(E)** Mobility of mobile electrons.

Figures 4A–E show the relationship between temperature and applied voltage.

In Figure 4A, it is found that the thickness of the device is negatively correlated with the rise in temperature. That is to say, the shorter the device is, the faster the temperature rises. Figure 4B illustrates the relationship between temperature and width of the band gap. When the width of the band gap is widest (0.46 eV in Figure 4B), the sudden increase occurs latest, but the change is most dramatic from 400 to 1,600°C. The curves of Figure 4C indicate that the relative permittivity of the material only affects the voltage value at which a sudden change of temperature occurs. What is noteworthy is that the two factors mentioned above have no influence on the final temperature value of the device (at around 2,100°C). Figure 4D presents the relationship between temperature relaxation time and temperature change. With the growth of temperature relaxation time, the temperature after the threshold voltage reaches much larger values. Compared with the temperature relaxation time, the curves in Figure 4E represent that the mobility of mobile electrons has the same impact on the temperature change. The only difference is that the rise in temperature is less dramatic.

We conclude the relationship between five factors and four characteristics illustrated in Table 2 (“↑” represents rise, while “↓” represents decline. “—” means irrelevant parameter).

**TABLE 2:** Relationship between device performance and its parameters.

	L ↑	ΔE ↑	ε↑	τ↑	μ↑
V_*th*_	**↑**	**↑**	**↑**	**↑**	**↓**
V_*h*_	**↑**	**↑**	**↑**	**↑**	**↓**
Selectivity	**—**	**↑**	**↑**	**↑**	**↑**
I_*OFF*_	**↓**	**↓**	**↓**	**—**	**↑**

It can be summarized that the thickness of the device and the width of the band gap have important roles in the threshold voltage on the switching. If the device is thinner, the threshold voltage will be lower and the off-state current will be larger. However, the thickness of the device makes no remarkable contributions on enhancing the selective. The width of the band gap and relative permittivity of the material have the same effect on the device performance. The analysis above demonstrates that with the increase of the two factors, threshold voltage and selectivity both rise and off-state current falls. When temperature relaxation time increases, threshold voltage and selectivity perform similarly to the two factors, but make no contribution to reduce off-state current.

## Application of Model

In this section, an OTS device with high selectivity and high threshold voltage is projected. On the basis of the analysis before, the width of the band gap and relative permittivity of the material are supposed to be larger while the mobility of the mobile electrons should be lower. Parameter values of five key factors are presented in Table 3.

**TABLE 3:** Parameter value of five key factors.

Symbol	Value	Units
Δ	0.8	eV
ε	18	
L	20 × 10^–9^	m
τ_*T*_	1 × 10^–14^	s
μ	4.9 × 10^–3^	m^2^ (Vs) ^–1^

We prepared a GeSe-based sample doping Sb according to the parameters in Table 3. Thereinto, GeSe thin film was 20 nm thick and was co-sputtered using the GeSe_2_ and Ge_2_Se_3_ targets via RF magnetron sputtering at room temperature. Besides, 40 nm-thick TiN electrodes were deposited using the DC reactive sputtering method with a TiN target. The sample was tested by positive DC sweep under 10 V.

**FIGURE 5:**
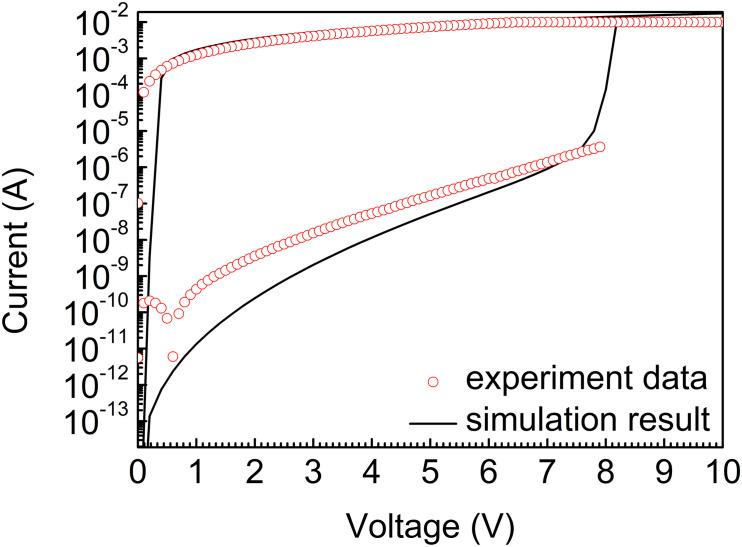
The experiment data of the sample (res dots) and simulation result (black line).

The experiment data of the sample (res dots) and simulation result (black line) are shown in Figure 5. The curves indicate that our model is able to match the experiment data approximately. It can be seen that threshold voltage, selectivity, and on-state current can be perfectly matched. The experiment data presents high selectivity characteristic at around 10^6^ and high threshold voltage characteristic at 8 V, which satisfy the expected design requirements. However, limited by device performance and test conditions, the matching results of hold voltage and off-state current deviate from the actual device. The simulation results demonstrate that a high selectivity and high voltage device is manufactured based on the analysis of our model parameters, which presents the validity of our model.

## Conclusion

Device models are crucial for device applications. In this article, a physically based model for threshold switching in OTS device was investigated. We provided a synthesis model based on Poole-Frenkel conduction mechanism accompanied by thermal dissipation effect for the first time. The thermal dissipation effect was able to improve the consistency between the model and the experimental sample. By comparing with the experimental data, the validity of this model was verified. The device model can provide a valuable tool for the compact physically based design of selector cells to manufacture high-performance selectors and to be utilized to design Hodgkin-Huxley neurons with a promising outlook.

## Data Availability Statement

The original contributions presented in the study are included in the article/supplementary material, further inquiries can be directed to the corresponding author/s.

## Author Contributions

SZ and BS conceived and designed the model and the experiments. BS and ZL provided assistance for the modeling work. SZ performed the simulation work and conducted the experiments. SZ, BS, ZL, SL, and QL contributed to the writing and editing of the manuscript. HX supervised the project. All authors discussed the results.

## Conflict of Interest

The authors declare that the research was conducted in the absence of any commercial or financial relationships that could be construed as a potential conflict of interest.
